# Supplementary material for: Development and Validation of a Novel Four Gene-Pairs Signature for Predicting Prognosis in DLBCL Patients
Source: Int J Mol Sci. 2024 Nov 28;25(23):12807. doi: 10.3390/ijms252312807 (PMC11640839; doi:10.3390/ijms252312807)
Supplement: Supplementary file 1 [file ijms-25-12807-s001.zip › Supplemental Materials/Figure S1.pptx]

## Slide 1
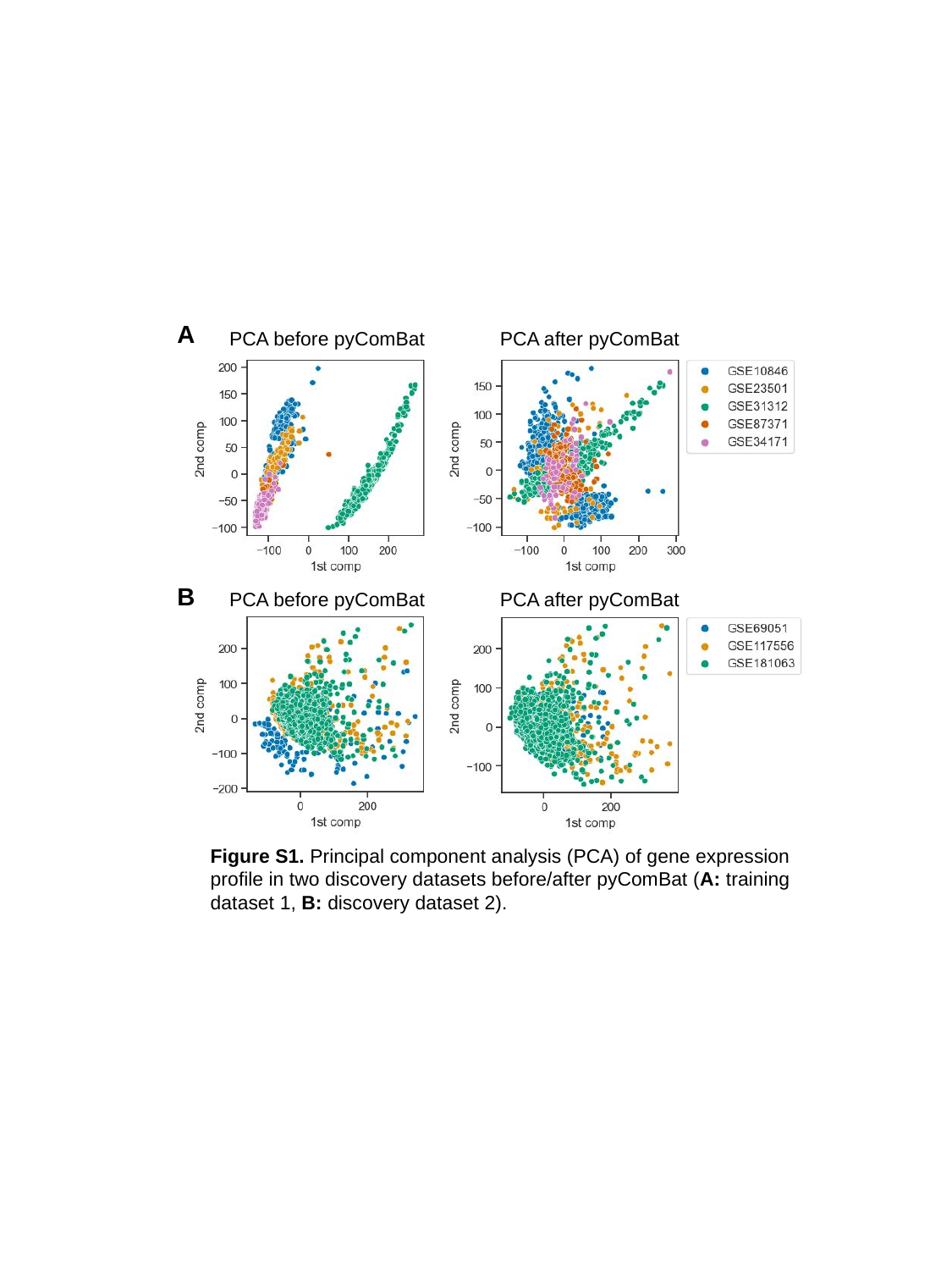

A
PCA before pyComBat
PCA after pyComBat
B
PCA before pyComBat
PCA after pyComBat
Figure S1. Principal component analysis (PCA) of gene expression profile in two discovery datasets before/after pyComBat (A: training dataset 1, B: discovery dataset 2).
