# Supplementary material for: Development and Validation of a Novel Four Gene-Pairs Signature for Predicting Prognosis in DLBCL Patients
Source: Int J Mol Sci. 2024 Nov 28;25(23):12807. doi: 10.3390/ijms252312807 (PMC11640839; doi:10.3390/ijms252312807)
Supplement: Supplementary file 1 [file ijms-25-12807-s001.zip › Supplemental Materials/Figure S2.pptx]

## Slide 1
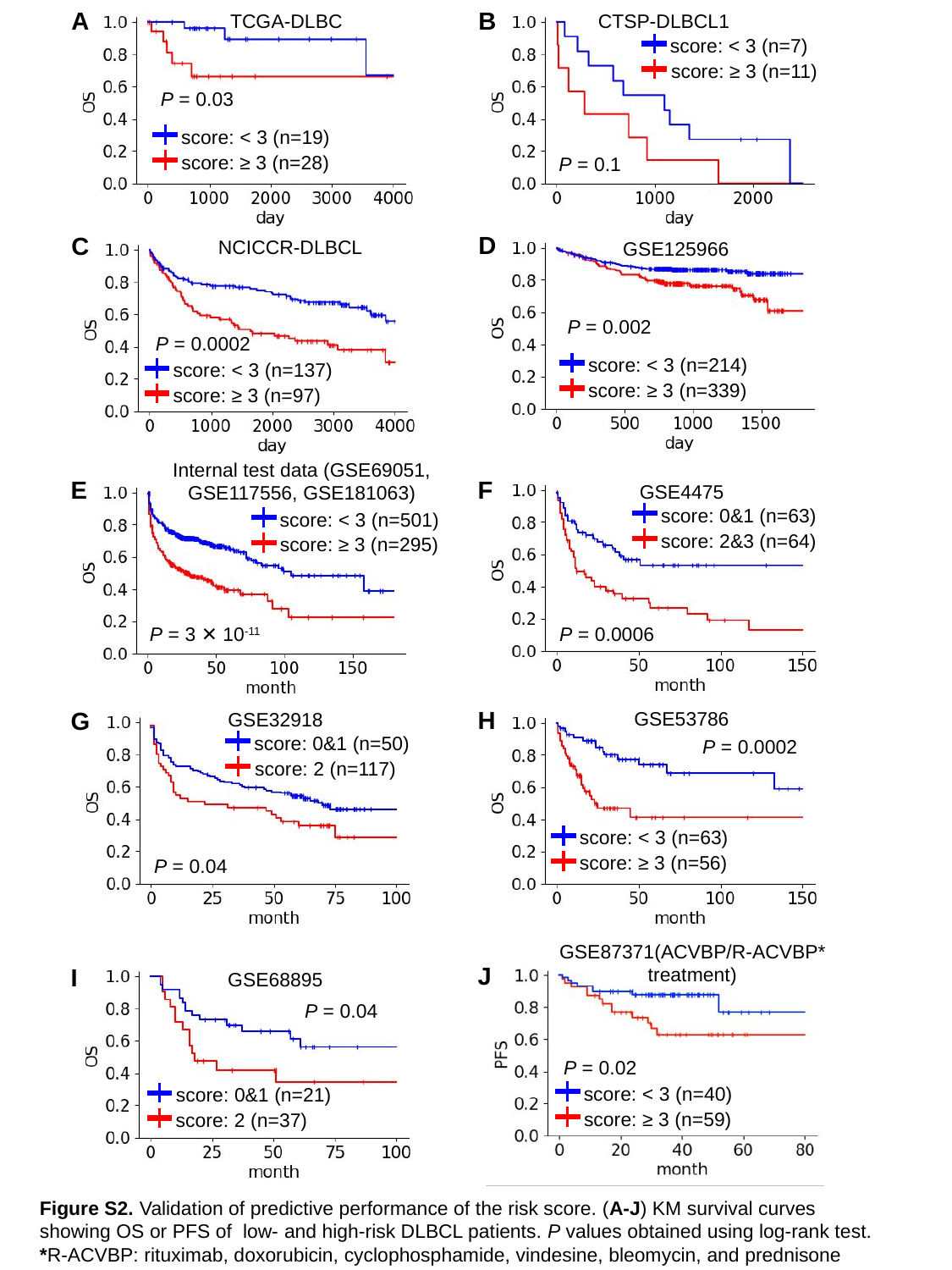

A
B
TCGA-DLBC
CTSP-DLBCL1
score: < 3 (n=7)
score: ≥ 3 (n=11)
P = 0.1
P = 0.03
score: < 3 (n=19)
score: ≥ 3 (n=28)
D
C
NCICCR-DLBCL
GSE125966
P = 0.002
score: < 3 (n=214)
score: ≥ 3 (n=339)
P = 0.0002
score: < 3 (n=137)
score: ≥ 3 (n=97)
Internal test data (GSE69051, GSE117556, GSE181063)
F
E
GSE4475
score: 0&1 (n=63)
score: 2&3 (n=64)
score: < 3 (n=501)
score: ≥ 3 (n=295)
P = 0.0006
P = 3 ✕ 10-11
H
G
GSE53786
GSE32918
score: 0&1 (n=50)
score: 2 (n=117)
P = 0.0002
score: < 3 (n=63)
score: ≥ 3 (n=56)
P = 0.04
GSE87371(ACVBP/R-ACVBP* treatment)
J
I
GSE68895
P = 0.04
P = 0.02
score: < 3 (n=40)
score: ≥ 3 (n=59)
score: 0&1 (n=21)
score: 2 (n=37)
Figure S2. Validation of predictive performance of the risk score. (A-J) KM survival curves showing OS or PFS of low- and high-risk DLBCL patients. P values obtained using log-rank test. *R-ACVBP: rituximab, doxorubicin, cyclophosphamide, vindesine, bleomycin, and prednisone
