# Supplementary material for: Development and Validation of a Novel Four Gene-Pairs Signature for Predicting Prognosis in DLBCL Patients
Source: Int J Mol Sci. 2024 Nov 28;25(23):12807. doi: 10.3390/ijms252312807 (PMC11640839; doi:10.3390/ijms252312807)
Supplement: Supplementary file 1 [file ijms-25-12807-s001.zip › Supplemental Materials/Figure S3.pptx]

## Slide 1
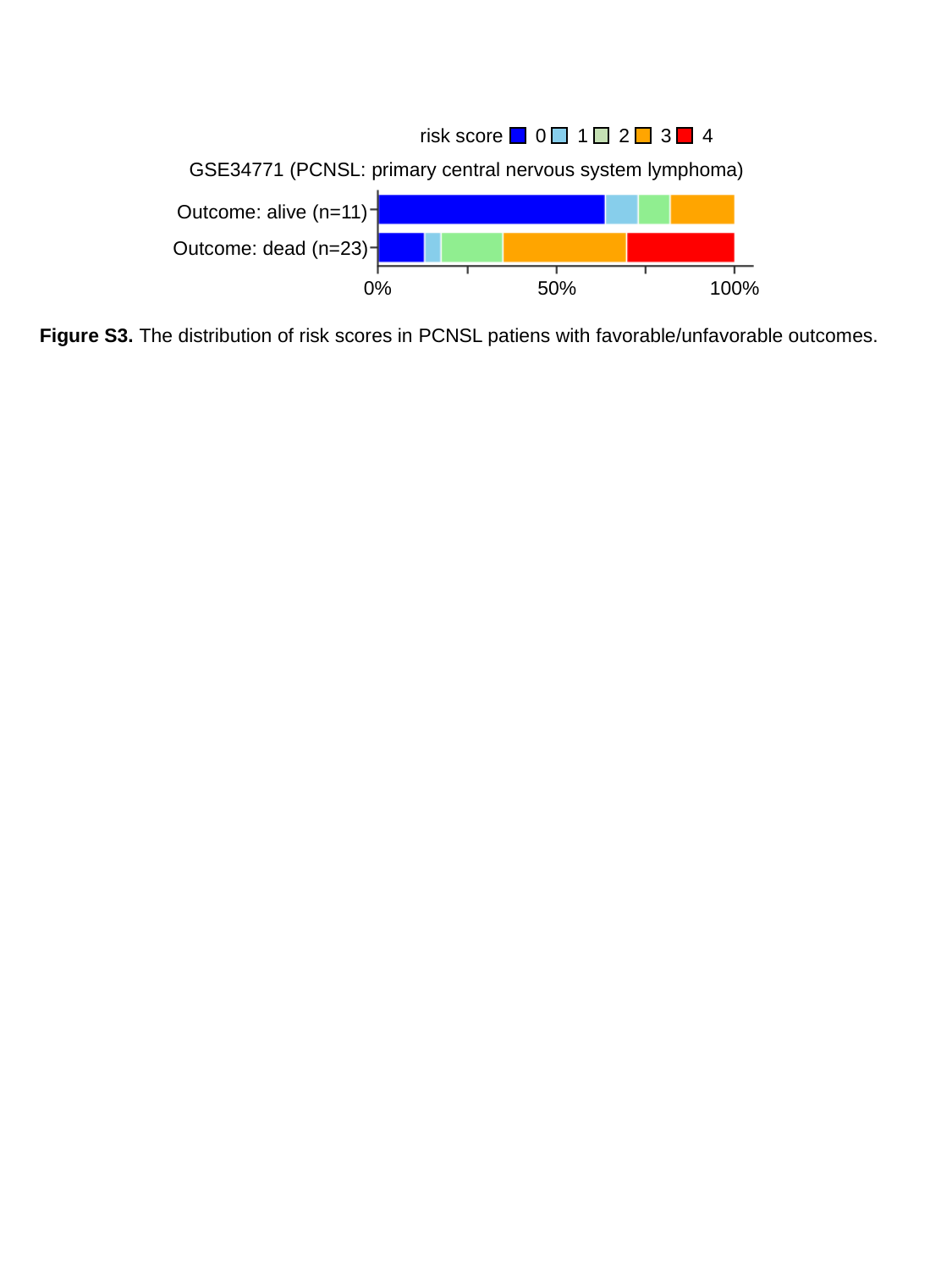

0
1
2
3
4
risk score
GSE34771 (PCNSL: primary central nervous system lymphoma)
Outcome: alive (n=11)
Outcome: dead (n=23)
0%
50%
100%
Figure S3. The distribution of risk scores in PCNSL patiens with favorable/unfavorable outcomes.
