# Supplementary material for: Development and Validation of a Novel Four Gene-Pairs Signature for Predicting Prognosis in DLBCL Patients
Source: Int J Mol Sci. 2024 Nov 28;25(23):12807. doi: 10.3390/ijms252312807 (PMC11640839; doi:10.3390/ijms252312807)
Supplement: Supplementary file 1 [file ijms-25-12807-s001.zip › Supplemental Materials/Figure S4.pptx]

## Slide 1
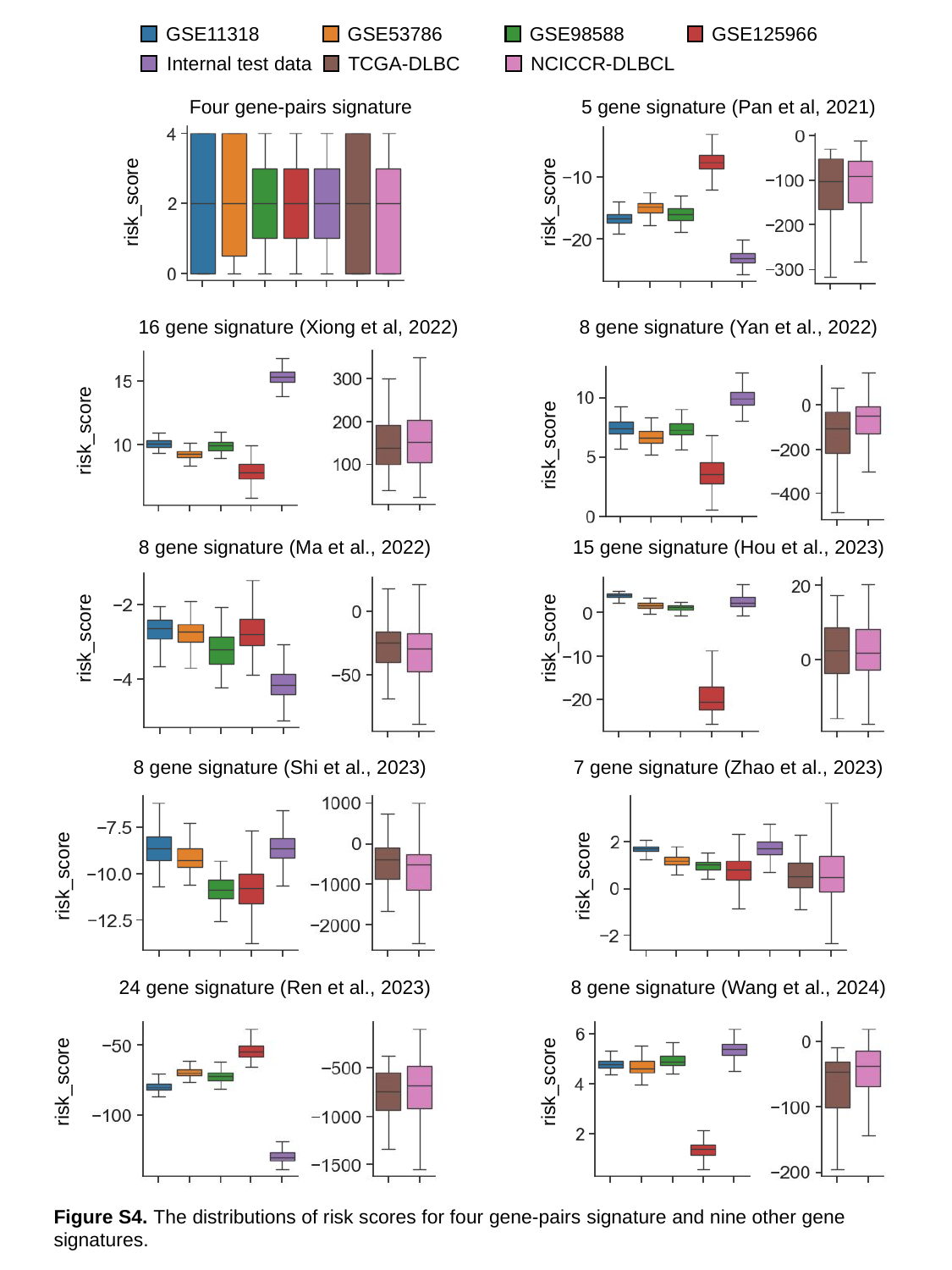

GSE11318
GSE53786
GSE98588
GSE125966
Internal test data
TCGA-DLBC
NCICCR-DLBCL
Four gene-pairs signature
5 gene signature (Pan et al, 2021)
risk_score
risk_score
16 gene signature (Xiong et al, 2022)
8 gene signature (Yan et al., 2022)
risk_score
risk_score
8 gene signature (Ma et al., 2022)
15 gene signature (Hou et al., 2023)
risk_score
risk_score
8 gene signature (Shi et al., 2023)
7 gene signature (Zhao et al., 2023)
risk_score
risk_score
24 gene signature (Ren et al., 2023)
8 gene signature (Wang et al., 2024)
risk_score
risk_score
Figure S4. The distributions of risk scores for four gene-pairs signature and nine other gene signatures.
